# Presence of Segmented Flavivirus Infections in North America

**DOI:** 10.3201/eid2608.190986

**Published:** 2020-08

**Authors:** Kurt J. Vandegrift, Arvind Kumar, Himanshu Sharma, Satyapramod Murthy, Laura D. Kramer, Richard Ostfeld, Peter J. Hudson, Amit Kapoor

**Affiliations:** Center for Infectious Disease Dynamics, Pennsylvania State University, University Park, Pennsylvania, USA (K.J. Vandegrift, P.J. Hudson);; Center for Vaccines and Immunity, The Research Institute at Nationwide Children’s Hospital, Columbus, Ohio, USA (A. Kumar, H. Sharma, S. Murthy, A. Kapoor);; Wadsworth Center, New York State Department of Health, Albany, New York, USA (L.D. Kramer);; University of Albany-SUNY School of Public Health, Albany (L.D. Kramer);; The Cary Institute for Ecosystem Science, Millbrook, New York, USA (R. Ostfeld);; Ohio State University College of Medicine and Public Health, Columbus (A. Kapoor)

**Keywords:** Alongshan, arbovirus, deer mice virome, flaviviruses, Jingmen tick virus, northeastern United States, synanthropic animals, tick-borne viruses, ticks, vectorborne infections, viral metagenomics, viruses, white-footed mouse, zoonoses

## Abstract

Identifying viruses in synanthropic animals is necessary for understanding the origin of many viruses that can infect human hosts and developing strategies to prevent new zoonotic infections. The white-footed mouse, *Peromyscus leucopus*, is one of the most abundant rodent species in the northeastern United States. We characterized the serum virome of 978 free-ranging *P. leucopus* mice caught in Pennsylvania. We identified many new viruses belonging to 26 different virus families. Among these viruses was a highly divergent segmented flavivirus whose genetic relatives were recently identified in ticks, mosquitoes, and vertebrates, including febrile humans. This novel flavi-like segmented virus was found in rodents and shares ˂70% aa identity with known viruses in the highly conserved region of the viral polymerase. Our data will enable researchers to develop molecular reagents to further characterize this virus and its relatives infecting other hosts and to curtail their spread, if necessary.

Most human infectious diseases have a zoonotic origin (*1*). RNA viruses are remarkable in their ability to evolve and infect a wide range of hosts, primarily due to their error-prone replication, small genome size, and ability to adapt (*2*). Recent studies describing human infections with animal coronaviruses and paramyxoviruses illustrate well the high zoonotic potential of animal RNA viruses (*3*). Not all viruses at the human–animal interface can breach the species barrier, and successful cross-species transmission often requires repeated introduction coupled with favorable circumstances (*3*). These multiple exposures provide increased opportunity for viral adaptation, and because humans are frequently exposed to viruses from synanthropic hosts, this channel becomes a likely route of infection. As such, we need to identify and characterize viruses from synanthropic animals to understand the origins of many human viruses and obtain insights into the emergence of potential zoonotic infections (*1*).

Rodents and bats are common sources of potential zoonotic viruses (*4*). In the northeastern United States, the white-footed mouse (*Peromyscus leucopus*) is one of the most widespread and abundant rodent species. These mice are highly adaptable, commonly become synanthropic, and invade human domiciles. Humans are exposed to a wide range of viruses from these mice, either directly as is the case with hantaviruses or indirectly through vectors such as ticks. These rodents are also known to harbor a range of zoonotic pathogens, including the tickborne *Borrelia burgdorferi*, which causes Lyme disease, and *Anaplasma phagocytilium*, which causes anaplasmosis (*5*). White-footed mice can also act as reservoirs for bacteria that are causative agents of Rocky Mountain spotted fever, tularemia, plague, and bartonellosis and for protozoans responsible for babesiosis, giardiasis, toxoplasmosis, and cryptosporidiosis (*6*). Infectious viruses reported in *P. leucopus* include lymphocytic choriomeningitis virus, Powassan or deer tick encephalitis virus, and hantaviruses (*7*,*8*).

The genome of flaviviruses is composed of a single-stranded positive-sense RNA that codes for a single polyprotein. The genome of Jingmen tick virus (JMTV), which was first identified in 2014 from ticks in the Jingmen province of China (*9*), is composed of 4 single-stranded positive-sense RNA segments, 2 of which encode a polymerase protein (NS5) and a helicase protein (NS3) that show close phylogenetic relatedness with the corresponding proteins of classical flaviviruses (*9*). Later, several genetically diverse relatives of JMTV were found in several species of ticks, insects, and mammals (*9*–*13*). Together, these JMTV-like viruses are highly diverse and show differences in the number of genomic segments as well as protein coding strategies (*9*,*10*,*12*). In 2018, a metagenomics study revealed the presence of JMTV-like sequences in serum samples from human patients with Crimean-Congo hemorrhagic fever in Kosovo (*14*). Two studies published in 2019 reported the presence of JMTV sequences in humans in China with febrile illness and a history of recent tick bites (*15*,*16*). To date, no information is available on the presence of JMTV infections in insects, ticks, or vertebrates in North America.

Knowledge about viruses infecting *P. leucopus* and the levels to which humans are being exposed is limited. Although several studies have examined the prevalence of hantaviruses (*17*) and highly diverse hepaciviruses (*18*), there has been no attempt to characterize the blood virome of these common rodents. We used an unbiased, metaviromics approach to identify all viruses in the serum samples of 978 free-ranging white-footed mice captured over a period of 7 years from suburban and wild areas of Pennsylvania.

## Materials and Methods

### Origin and Details of White-Footed Mouse Samples

We collected serum samples from *P. leucopus* mice each spring and autumn during 2011–2017 from 4 sites in central Pennsylvania. We selected these sites because they provided gradient levels of human activity and so exposure to rodents; site 1 had the highest level of exposure and site 4 had the lowest. At site 1 (Deer Pens), mouse infestation is reported in residences, workplaces, and the surrounding area, providing a potentially high level of exposure to rodents. Site 2 (Spray Fields) is a disturbed forest where treated wastewater is sprayed; it is frequented daily by maintenance employees and dog walkers, but there are no residences on site. Site 3 (Scotia) is on Pennsylvania state game lands that border housing communities and has mostly seasonal visitors, such as hikers, hunters, and trappers. Site 4 (Stone Valley) is in a large contiguous forest that has very few people or residences. Additional details about the sites have been described previously (*19*).

Whole blood was obtained from the retro-orbital sinus of the anesthetized mice. These blood samples were centrifuged at 8,000 × *g* for 10 min and the serum was then collected with a micropipette. Samples were stored at −80°C. All procedures were approved by Penn State’s Institutional Animal Care and Use Committee (IACUC #46246).

### Metagenomics, Metaviromics, and Bioinformatics

We used serum samples from 978 wild free-ranging *P. leucopus* mice to generate the high-throughput sequence data to characterize the virome of these rodents. We used a 10-µL subsample of serum from each individual mouse to create 9 pools. We assigned individual mice to pools by bodyweight (as a proxy for age) and study site. We centrifuged serum pools at 8,000 × *g* for 10 min to remove particulate matter. The supernatants were treated with DNase (100 U), RNase (20 U), and Benzonase (250 U) to enrich samples for particle-protected (virion) nucleic acids. We used the QIAamp Viral RNA Mini Kit (QIAGEN Inc., https://www.qiagen.com) to extract nucleic acid from the serum pools and eluted it in 60 μL of elution buffer supplemented with 40 U of ribonuclease inhibitor, then stored at it −80°C before library preparation. We used a unique 20-nt barcoded oligonucleotide primer for each sample pool during reverse transcription PCR (RT-PCR) and second-strand DNA synthesis, as previously described (*20*). We prepared libraries for Illumina sequencing as previously described (*21*) and performed sequencing on a HiSeq 4000 platform (Illumina Inc., https://www.illumina.com) for 2 × 150 cycles in the Biomedicine Genomics Core at the Research Institute of Nationwide Children’s Hospital (Columbus, Ohio, USA).

We used the 20-nt unique barcodes included in the primers to make libraries to demultiplex the sequence data. After removing low-complexity regions and low-quality bases, we aligned paired-end reads to the *P. leucopus* genomes with Bowtie2 mapper version 2.0.6 (SourceForge, https://sourceforge.net) to remove the host-derived sequences. We used MIRA version 4.0 (SourceForge) (*22*) for de novo assembly of remaining sequences. Finally, we analyzed all contigs and unique reads using MegaBLAST https://blast.ncbi.nlm.nih.gov/Blast.cgi) against the GenBank nonredundant nucleotide database. Next, we used BLASTX to analyze sequences that showed poor or no homology (e-score >0.001) against the viral GenBank protein database, followed by BLASTX against the nonredundant protein database. We then used reference genomes of known viruses available in GenBank to extract related sequences present in the 9 serum pools. Finally, we used specific PCR assays and traditional dideoxy sequencing of amplicons to confirm bioinformatics-based assembly of virus reads.

### Presence and Prevalence of Flavi-Like Segmented Virus and South Bay Virus

We selected 2 viruses recently identified in ticks for further characterization: a highly divergent JMTV-like virus, provisionally named flavi-like segmented virus (FLSV or FLSV-US); and South Bay virus (SBV), a tick virus that belongs to the family *Nairoviridae*, order *Bunyavirales* (*23*,*24*). We extracted individual serum samples from the 72 P. leucopus mice that made up the pool with the maximum number of unique FLSV-US sequence reads and screened for the presence of FLSV-US RNA by using a nested RT-PCR targeting the conserved NS5 polymerase region. We used the primers FLSV-US-F1 (5′-GGWGCYATGGGYTACCAGAT-3′) and FLSV-US-R1 (5′-TCCARGGTGAGTARTCCTTTCG-3′) in the first round of PCR, and FLSV-US-F2 (5′-GGWGCYATGGGYTACCAGATGGA-3′) and FLSV-US-R2 (5′-CCARGGTGAGTARTCCTTTCGARATC-3′) in the second round. The first-round PCR cycle included 8 min of initial denaturation at 95°C; 10 cycles of 95°C for 40 s, 56°C for 1 min, and 72°C for 1 min; 30 cycles of 95°C for 30 s, 54°C for 30 s, and 72°C for 1 min; and a final extension at 72°C for 5 min. In the first 10 cycles, the annealing temperature was ramped down by 0.5°C each cycle to enable nucleotide mismatch tolerance during primer hybridization. The second-round PCR conditions included 8 min of initial denaturation at 95°C; 10 cycles of 95°C for 40 s, 60°C for 1 min, and 72°C for 1 min; 30 cycles of 95°C for 30 s, 58°C for 30 s, and 72°C for 1 min; and a final extension at 72°C for 5 min.

We used a heminested RT-PCR targeting the viral polymerase gene to screen serum samples from the same 72 mice for the presence of SBV. We used the primers SBV-F1 (5′-AYCCAGATTGGAARCACTTCATAATG-3′) and SBV-R1 (5′-CCATATGTDGTAATMACYTTWGCATA-3′) for first round of PCR, and SBV-F2 (5′-GTTATGTTGAAGGACCTTAACAAAG-3′) and SBV-R1 for the second round. The PCR cycle for both rounds of PCR included 8 min of initial denaturation at 95°C; 10 cycles of 95°C for 30 s, 55°C for 2 min, and 72°C for 1 min; 30 cycles of 95°C for 25 s, 54°C for 30 s, and 72°C for 30 s; and a final extension at 72°C for 5 min. In the first 10 cycles, the annealing temperature was ramped down by 0.5°C each cycle to enable nucleotide mismatch tolerance during primer hybridization step.

### Phylogenetic Analysis and Genome Organization of FLSV-US

We aligned sequences that showed substantial similarity with JMTV NS5 proteins with reference sequence YP_009029999.1 (GenBank accession no. MN811583) to design PCR primers for direct amplification of 2,568-nt FLSV-US sequences. After we confirmed the FLSV-US assembled sequence by Sanger sequencing, we aligned the translated protein sequence with the sequence of known JMTV-like viruses (aa 55 to 913 of NS5 protein) using BLOSUM protein weight matrix, using default parameters in MEGA 7.1 (*25*). We constructed a phylogenetic tree using the maximum-likelihood method and the best pattern substitution model with the lowest Bayesian information criterion score, LG+G+I model (Le Gascuel, Gamma distribution with 5 rate categories and evolutionary invariable sites) (*26*). To confirm that the FLSV-US genome is segmented, we used FLSV-US reads showing substantial similarity to JMTV NS3 protein for acquiring the 3′ end of FLSV-US NS3 coding segment using a poly-T oligonucleotide-primed cDNA synthesis followed by specific PCR, as previously described (*27*). The 3′ end sequence of FLSV-US NS3 coding segment and the complete 3′ untranslated region (UTR) is available in GenBank (accession no. MN811584).

## Results

### Serum Virome of *P. Leucopus*

**Figure 1:**
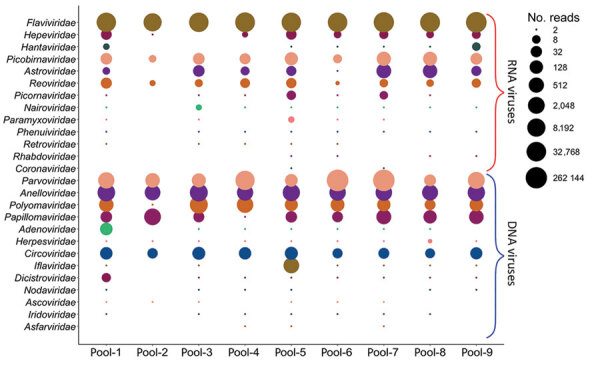
Bubble plot showing the abundance of different viruses in the serum virome of the white-footed mouse. Sequence reads showing the highest sequence similarity to known viruses were normalized as reads per million and were grouped into RNA and DNA virus families. Read numbers were transformed to log2, where the cutoff is ≥2 reads, represented by the smallest circle.

A total of 242 million paired-end sequences were generated from the 9 pools representing the serum samples of 978 *P. leucopus*. Of these, 148 million (61%) were derived from the host genome. Of the remaining 94 million sequences, 65.6% of reads showed substantial similarity to known viruses (E-value <0.001). Further analysis classified these sequences into 26 known RNA and DNA virus families (Figure 1).

### DNA Virome

We found parvoviruses, circoviruses, Torque teno viruses (TTV), polyomaviruses, and papillomaviruses in all 9 serum pools, and these were the most abundant DNA viruses. Among parvoviruses, bocaparvovirus (BocPV) sequences shared up to 90% aa identity with the nonstructural protein of a rodent BocPV identified in Brazil (*28*), and adeno-associated viruses (AAV) shared up to 65% aa identity with the nonstructural protein of a caprine AAV (*29*). The identified TTV sequences were genetically equidistant from rodent TTV (GenBank accession no. AEF5869) and mosquito TTV (GenBank accession no. AEF58766) (*30*); the sequences shared up to 60% aa identity with these 2 species of the genus *Omegatorquevirus* (*31*). The polyomavirus sequences shared up to 79% aa identity with the large T antigen of a polyomavirus isolated from the Montane grass mouse (*Akodon montensis*) (*32*). These papillomavirus sequences belong to a tentatively identified new virus species within the genus *Iotapapillomavirus* that shares up to 79% aa identity with the papillomavirus found in *P. maniculatus* (GenBank accession no. NC_039039) (*33*).

### RNA Virome

The most abundant RNA viruses present in the *P. leucopus* serum pools were the genetically diverged variants of hepaciviruses and pegiviruses (*34*). Phylogenetic analyses of these sequences indicated that the *P. leucopus* hepaciviruses form a distinct new genetic cluster (*35*). We found sequence reads of a novel hepevirus in 8 of the 9 serum pools. Genetic analysis of these *P. leucopus* hepevirus sequences suggest it is a new member of the genus *Orthohepevirus* because it shares <75% aa identity with the capsid proteins of hepevirus isolated from the common kestrel, *Falco tinnunculus* (*36*), and from rodents (*37*). Astrovirus-like sequences found in 8 serum pools shared up to 64% aa identity in the capsid protein and up to 73% aa identity in the nonstructural protein with the known species of the genus *Mamastrovirus*. Three of the 9 serum pools had sequences that were genetically closest to bat astroviruses (*38*) but shared up to 61% aa identity in the nonstructural protein. Sequences sharing up to 97% aa identity with hantaviruses recently found in *Peromyscus* sp. were also present in 6 serum pools (*39*). Coronavirus-related sequences were found in 2 serum pools and shared 98% aa identity with the porcine hemagglutinating encephalomyelitis virus (GenBank accession no. ACH72649). Paramyxovirus sequences were present in 5 serum pools and shared up to 90% nt identity with paramyxoviruses isolated from 3 rodent species: *Apodemus peninsulae* (accession no. KY370098), *Rattus norvegicus* (accession no. KX940961), and *R. andamanensis* (accession no. JN689227).

Rotavirus sequences were present in all serum pools and shared up to 78% nt identity in the NS3 gene with bovine rotavirus (GenBank accession no. JX442794) and human rotavirus (accession no. AB748601); <74% nt identity in the VP2 gene with avian rotavirus (accession no. FJ169854), human rotavirus (accession no. KF035108), and canine rotavirus (accession no. LC326510); and <45% aa identity in the VP6 gene to that of human rotavirus (accession no. AHK24897) and canine rotavirus (accession no. ACH97443). Picornavirus sequences were detected in 7 serum pools; these sequences shared up to 65% aa identity with marmot cardiovirus (accession no. AVX29481), <48% aa identity with rodent enterovirus (accession no. YP_009508417), <67% aa identity with *Rattus tanezumi* hunnivirus (accession no. AWK02689), and <87% aa identity with rodent hepatovirus (accession no. ALL35326).

### SBV

Analysis of virome data showed the presence of SBV sequences in 8 of the 9 *P. leucopus* serum pools. Because SBV is not known to infect vertebrates, we used RT-PCR to screen serum samples of 72 individual mice; 5 samples were positive for SBV. Subsequent sequencing of PCR products showed that the SBV variants present in mice serum samples share 99%–100% nt identity with the SBV variants identified in *Ixodes scapularis* black-legged ticks (*24*,*40*).

### FLSV 

We found 370 sequence reads that showed the highest sequence similarity with the NS5 proteins of JMTV-like viruses. Similarly, we found 42 sequence reads that showed the highest sequence similarity with the NS3 proteins of JMTV-like viruses (*9*,*11*,*12*,*15*). These FLSV-US sequences were genetically equidistant from the 3 prototypic virus members of the JMTV-like virus group, namely JMTV, Mogiana tick virus, and Alongshan virus (*15*,*16*,*24*). Considering the potential consequentiality of a divergent JMTV-like virus infection in a widely distributed mammalian species in North America, we developed a broadly reactive PCR assay to confirm our results and to define infection prevalence of FLSV-US in *P. leucopus* mice.

**Figure 2:**
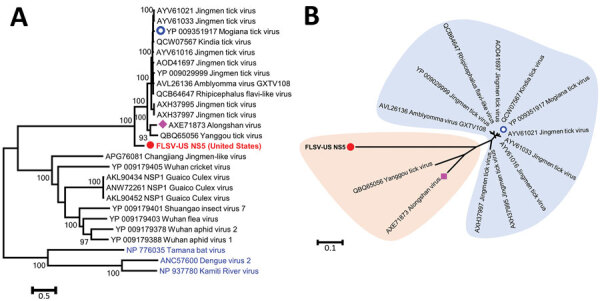
Phylogenetic analyses of FLSV-US (red) on the basis of NS5 proteins corresponding to amino acid positions 55 to 913 on Jingmen tick virus reference sequence YP_009029999.1. The trees are drawn to scale, with branch lengths measured in the number of substitutions per site. A) Phylogenetic analysis of conventional flaviviruses (blue) and recently identified segmented flavi-like viruses from ticks, mosquitoes, and other arthropods. B) Phylogenetic analysis of viruses closely related to FLSV-US. Alongshan virus was found in patients from China (purple diamond), Mogiana tick virus was found in ticks from Brazil (blue shading), and all other viruses were found in ticks collected in China. FLSV, flavi-like segmented virus.

PCR screening and amplicon sequencing confirmed the presence of FLSV-US nucleic acids in serum samples from 8 of the 72 mice in this pool, indicating an infection prevalence of 11%. Comparative sequence analysis determined ≈3.8% nt divergence in the NS5 region among FLSV-US variants infecting these mice (data not shown); all of these mutations were synonymous. Next, we acquired the near-complete coding region for a FLSV-US NS5 polymerase segment and used it for phylogenetic analysis (Figure 2). We determined that FLSV-US is more genetically diverse than the previously identified JMTV variants and Alongshan virus and shared ˂70% aa identity with these viruses (Table). We used a poly-T oligonucleotide primed cDNA synthesis to acquire the 3′ end of FLSV-US NS3 coding segment. Sequencing of the amplicon revealed the presence of a 388-nt 3′ untranslated region preceded by 96-aa NS3 protein coding region that showed highest sequence similarity with the carboxy terminal of JMTV NS3 protein. These results confirm that the FLSV-US genome is segmented like other known JMTV-like viruses and that the FLSV-US NS3 protein coding segment is polyadenylated.

**Table:** Percentages of pairwise amino acid distances between NS5 protein of FLSV-US and known JMTV-like viruses on the basis of the NS5 protein region corresponding to aa positions 55 to 913 on JMTV reference sequence YP_009029999.1

Sequence	ID*	A†	B	C	D	E	F	G	H	I	J	K	L	M	N
FLSV-US NS5†	A														
AOD41697 JMTV	B	31.2													
AVL26136 AMBV	C	31.8	2.8												
AXE71873 ALSV	D	34.7	19.6	19.8											
AXH37995 JMTV	E	34.7	7	7.3	19.4										
AXH37997 JMTV	F	32.2	6.9	7.2	19.4	0.1									
AYV61016 JMTV	G	31.5	2.8	2.9	19	6.8	6.6								
AYV61021 JMTV	H	31.8	4	4.3	19.4	7.3	7.2	3.1							
AYV61033 JMTV	I	31.9	4.1	4.4	19.3	7.2	7.1	3.3	0.6						
QBQ65056 YTV	J	33.4	20.4	20.5	18.3	20.1	20.1	20.1	20	19.9					
QCB64647 RFLV	K	31.5	2.4	1.5	19.1	6.4	6.3	2.3	3.4	3.5	19.9				
QCW07567 KTV	L	31.7	3.5	3.6	19.4	7.2	7.1	2.6	2.9	3	20.4	3			
YP_009029999 JMTV	M	31.8	3.7	2.3	19.6	7.2	7.1	3.4	4.4	4.4	20.4	2.2	3.7		
YP_009351917 MTV	N	30.8	3.7	3.8	19.7	6.9	6.8	2.4	2.7	2.8	20.4	3.5	2.7	4.2	

ALSV, Alongshan virus; AMBV, Amblyomma virus; ID, sequence identifier; JMTV, Jingmen tick virus; KYV, Kindia tick virus; MTV, Mogiana tick virus; RFLV, Rhipicephalus flavi-like virus; YTV, Yanggou tick virus. †Virus identified in Pennsylvania, USA, as part of this study. *Identifiers A–N in the top row represent the corresponding A-Z sequences in the left-hand column.

## Discussion

Synanthropic small mammals serve as reservoirs for many zoonotic infections (*41*–*46*). Mice of the genus *Peromyscus* are highly adaptable and thrive in human-modified landscapes. In particular, *P. leucopus* and *P. maniculatus* are closely related and very abundant in North America, with *P. leucopus* most common in the eastern two thirds of the United States, plus Canada and northern Mexico, and *P. maniculatus* present in the central and western United States. These rodents have been recognized as reservoirs of highly pathogenic hantaviruses, but the diversity of viruses infecting them has remained largely unknown. A recent study (*18*) showed the presence of highly diverse hepaciviruses and pegiviruses in these rodents, and a study published in 2011 by Phan et al. identified several other viruses in fecal samples of 20 *P. leucopus* mice (*47*). Our study expands the host range of the recently identified tick virus SBV and identifies a highly diverse virus, FLSV-US, whose genetic relatives were recently shown to be pathogenic in humans (*15*,*16*).

Our results show high genetic diversity among viruses infecting these rodents. We not only confirmed infections of hantaviruses, hepaciviruses, and pegiviruses but also found viruses representing homologs of almost all viruses commonly present in human serum samples: anelloviruses, parvoviruses, polyomaviruses, papillomaviruses, and hepatitis E virus. We also found several viruses not commonly present in human or animal serum samples, such as coronaviruses, paramyxoviruses, astroviruses, enteroviruses, and rotaviruses. It is plausible that some mice, when captured, had transient viremia of these otherwise respiratory or gastroenteric infections. It would also be worthwhile to further investigate other samples including feces, skin, urine, saliva, and tissue or organs to identify additional viral diversity. This may also aid in determining the tissue tropism of these viruses and provide clues to potential routes of transmission.

Recently, several novel viruses have been identified in ticks from the United States (*24*). However, the host range and public health relevance of most of these new tick viruses remains unknown (*24*). We found 1 of these newly identified and highly prevalent tick viruses, SBV, in *P. leucopus* serum samples. To the best of our knowledge, SBV has not been detected in a vertebrate host; thus, our study indicates that this new tick virus can infect mammals and may have a wider host range than was previously known. Comparative sequence analyses indicate that the SBV variants detected in *P. leucopus* serum samples share 99%–100% nt identity with the SBV variants identified in ticks, indicating their common origin.

Several recent studies to characterize the viromes of ticks and mosquitoes in North America showed an absence of JMTV-like viruses (*24*,*40*,*48*). The presence of FLSV infections in a rodent species that is also a common host of ticks and mosquitoes is therefore intriguing and raises questions about the source of FLSV- infection in these mice. It is plausible that FLSV-US transmits through an arthropod vector other than ticks or mosquitoes because some distantly related viruses were found in pools of insects and arachnids (*10*). 

In conclusion, we detected FLSV infections in a widespread mammalian species in North America, which is important because distant genetic relatives of FLSV-US have been shown to be transmitted by ticks and mosquitoes (*9*,*13*) and readily able to infect humans (*15*,*16*). Until recently, these unusual flavi-like viruses had only been found in ticks, mosquitoes, and animals from China, Kosovo, and Brazil (*11*,*14*,*16*). However, in 2019, Wang et al. reported JMTV viremia in 86 of 374 humans with febrile illness, headache, and history of tick exposure (*16*). In addition, JMTV was shown to replicate in several cell lines of human and animal origin (*15*,*16*). Taken together, these studies indicate that FLSV-US and related viruses have the potential to infect a wide range of mammals, including humans. Finally, FLSV-US is genetically distinct from all known viruses; therefore, its sequence data will help in the identification of FLSV-US and its related variants infecting animals and humans in North America.
